# Cardioprotective Potential of Grape-Derived Polyphenols in Cancer Therapy-Related Cardiotoxicity

**DOI:** 10.3390/ijms27188095

**Published:** 2026-09-11

**Authors:** Assiya Maikenova, Assel Urazbayeva, Zhuldyz Myrzabay, Nazym Askambayeva, Alexander Gulyayev, Alexander Digai, Ainur Tauekelova, Ayaulym Nurgaziyeva, Zhanel Mukhanbetzhanova, Mohamad Aljofan

**Affiliations:** 1 National Laboratory Astana, Center for Life Sciences, Nazarbayev University, Astana 010000, Kazakhstan; assiya.maikenova@nu.edu.kz (A.M.); assel.urazbayeva@nu.edu.kz (A.U.); akin@mail.ru (A.G.); anurgozhina@nu.edu.kz (A.N.); zhanel.pernebek@nu.edu.kz (Z.M.); 2 Department of Biomedical Sciences, Nazarbayev University School of Medicine, Astana 010000, Kazakhstan; zhuldyz.myrzabay@nu.edu.kz (Z.M.); nazym.askambayeva@nu.edu.kz (N.A.); 3 National Research Oncology Center, Astana 010000, Kazakhstan; aleksandr_digai@mail.ru; 4 Heart Center, Corporate Fund “University Medical Center”, Astana 010000, Kazakhstan; ainuratau@mail.ru

**Keywords:** polyphenols, cardiotoxicity, oxidative stress, breast cancer, biomarkers, antioxidant activity, flavonoids, chemotherapy, doxorubicin

## Abstract

Chemotherapy-induced cardiotoxicity is a serious consequence of chemotherapy during cancer treatment that can affect any patient. The purpose of this review is to highlight the development of cardiotoxicity associated with the use of anthracyclines or trastuzumab and to examine prevention and treatment methods using polyphenols, biologically active substances that hold significant therapeutic potential. The main mechanisms of this pathology, modern biomarkers for detection, and the classification and biological activity of polyphenols are discussed. Emphasis is placed on polyphenols found in grapes and their antioxidant potential as a protector of the cardiovascular system.

## 1. Introduction

The World Health Organization (WHO) reported that approximately 2.3 million people were diagnosed with breast cancer worldwide in 2022, resulting in approximately 670,000 deaths [1]. Moreover, breast cancer was the most commonly diagnosed cancer, more than twice as common as lung cancer, which ranked second in the list of the 5 most common oncological diseases in women that same year [1]. Breast cancer occurs worldwide, but the incidence of the disease varies from country to country.

According to GLOBOCAN 2024, Asia accounted for 45.1% of newly diagnosed breast cancer cases and 48.3% of breast cancer deaths worldwide [2]. In the Republic of Kazakhstan, breast cancer ranks first among malignant neoplasms in women. On average, up to 5000 patients with breast cancer are diagnosed in the country annually, and up to 1200 women die from this disease [3,4]. The incidence of breast cancer continues to rise worldwide. The future prognosis for 2050 made by Kim et al. states that new registered cases of malignancy and subsequent deaths will increase significantly by 38% and 68%, respectively. This increase is expected to disproportionately affect women living in developing countries [5].

Therapy for breast cancer typically includes anthracycline-based chemotherapy and HER2-targeted agents such as trastuzumab, both of which have been found to effectively improve survival outcomes.

However, despite their clinical benefits, breast cancer treatments are associated with an increased risk of cardiovascular toxicity. Anthracyclines are well known for causing severe cardiovascular effects collectively referred to as cardiotoxicity [6]. This is a general term that describes toxicity that can exert direct (e.g., damage to heart structure) or indirect (e.g., coagulation abnormalities) effects on cardiac function. In addition, this condition can occur at the beginning, during or even after successful cancer treatment. In addition, trastuzumab is also associated with an increased risk of cardiotoxicity, although the underlying mechanisms differ. HER2 (human epidermal growth factor receptor 2)-targeted agents cause reversible left ventricular dysfunction, particularly when administered with or after anthracyclines. Cardiotoxicity may develop immediately during treatment or months to years after therapy completion, thereby reducing quality of life by increasing cardiovascular morbidity and mortality. Therefore, the clinical focus has expanded beyond breast cancer treatment alone to include strategies for the prevention and mitigation of cardiotoxicity and its associated manifestations.

One such strategy might be the administration of polyphenols, which are well known for their potent antioxidant, anti-inflammatory, anticancer, and cardioprotective properties. Among them, grape-derived polyphenols are of particular interest, including catechins, anthocyanins, resveratrol, and proanthocyanidins, which have demonstrated beneficial effects in in vitro and in vivo experimental models of oxidative stress, endothelial dysfunction, mitochondrial injury, and cardiac remodeling.

Because the source and manufacturing origin of individual polyphenols were not consistently reported across the literature, compounds were classified according to the experimental material described in each study rather than assuming their botanical origin. Accordingly, studies using grape extracts or other grape-derived materials were identified separately from those investigating individual polyphenols that are naturally occurring constituents of grapes.

This review focuses on grape-derived products and associated polyphenols in the context of cardioprotection. This work integrates and links the potential effects of polyphenols with the molecular mechanisms underlying cardiotoxicity associated with anticancer therapy and examines supporting data on cardioprotection obtained in other cardiovascular models. Furthermore, the discussion of the mechanisms of cardiotoxicity is complemented by information on a modern panel of biomarkers used to detect and assess myocardial injury. In addition to mechanistic data, the review addresses the pharmacokinetics, bioavailability, safety, and potential impact of grape polyphenols on the efficacy of anticancer therapy. Thus, the review provides an applied perspective on the potential use of grape-derived polyphenols in cardio-oncology.

Therefore, this review aims to evaluate the cardioprotective potential of grape-derived polyphenols against cancer therapy-induced cardiotoxicity and to assess their relevance to supportive strategies in oncology treatment.

## 2. Methodology

A targeted literature search was conducted to identify studies relevant to the cardioprotective potential of grape-derived polyphenols. The literature search was completed on 31 August 2026, which was defined as the last day for the inclusion of publications. Studies published between 2000 and 31 August 2026 were considered for inclusion. Relevant publications were identified through searches of PubMed, Scopus, and Google Scholar using combinations of keywords including grape, *Vitis vinifera*, grape extract, grape pomace, grape seed, grape skin, polyphenols, resveratrol, quercetin, anthracyclines, trastuzumab, cardiotoxicity, cardiac dysfunction, oxidative stress, breast cancer, antioxidant activity, cardioprotection, anticancer, antitumor, tumor, proanthocyanidins, doxorubicin. Search terms were combined using the Boolean operators AND and OR. For example, (“grape extract” OR “grape pomace”) AND (“doxorubicin-induced-cardiotoxicity” OR cardioprotection). Different combinations of these and the other keywords listed above were used during the literature search. In addition, the literature search was supplemented by screening the reference lists of relevant publications to identify additional important studies. Priority was given to publications from the preceding five years; however, older studies such as those from the 2000s were included when they provided important foundational, mechanistic, or clinical evidence. The selected works are primarily focused on the topic of the present review. However, studies investigating other disease models were also considered if they provided relevant evidence regarding the biological properties and mechanisms of action of polyphenols. Studies using commercially obtained polyphenolic compounds were also included when the investigated compounds were naturally occurring constituents of grapes or grape-derived materials. The exclusion criterion applied to studies focusing on polyphenols or other bioactive compounds that are not naturally occurring constituents of grapes. However, studies investigating other bioactive compounds were not considered unless these substances were used as comparison treatments in studies evaluating grape-derived products or grape-associated polyphenols.

Publications for Table 1 and Table 2 were selected from the literature identified during the search described above. Studies were included in Table 1 when they specifically described the protective effects of grape-derived products or grape-associated polyphenols against cancer therapy-associated cardiotoxicity. Table 2 includes studies investigating the cardioprotective effects of grape-associated polyphenols in broader cardiovascular and experimental settings and provides supporting evidence.

## 3. Mechanisms of Chemotherapy-Induced Cardiotoxicity

The use of many anticancer drugs is limited by their adverse effects, particularly cardiovascular complications collectively referred to as cardiotoxicity. This term refers to a chemotherapy-induced decrease in the ejection fraction (EF) of the left ventricle (LV) due to LV hypokinesia, leading to symptoms of chronic heart failure (CHF) [7]. In addition to systolic and/or diastolic myocardial dysfunction and CHF, cardiotoxicity includes ischemic heart disease (IHD), arterial hypertension (AH), pulmonary hypertension, strokes, pericarditis, valvular lesions, arrhythmias, thrombosis and thromboembolism, and bleeding. All these complications have an extremely unfavorable effect on the prognosis of cancer patients. Consequently, cardiovascular complications have become one of the leading causes of non-cancer-related morbidity and mortality among cancer survivors [8].

**Figure 1:**
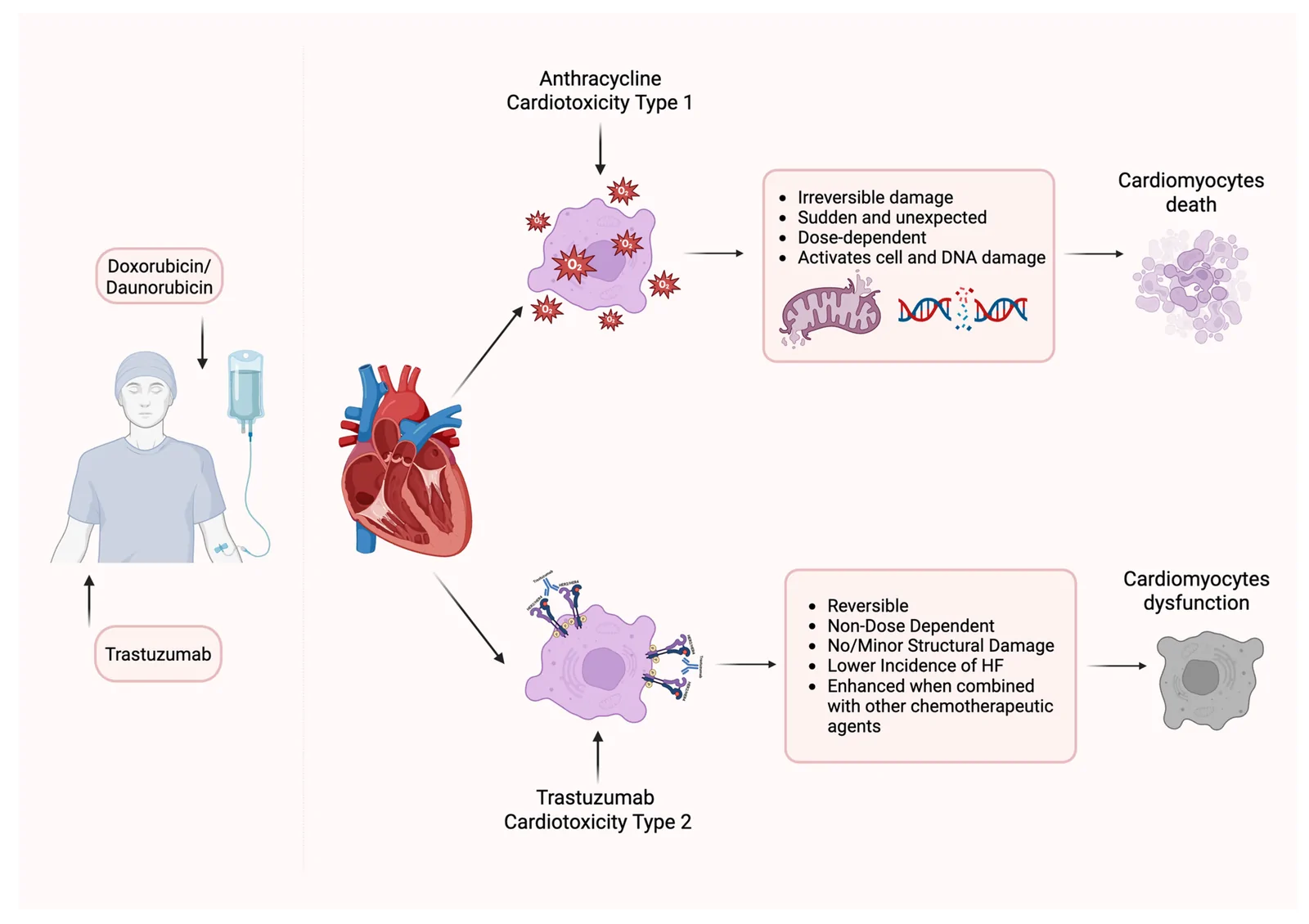
Historical Type I and Type II classification of cancer therapy-related cardiac toxicity associated with anthracyclines and trastuzumab (Created in BioRender. Maikenova, A. (2026), https://BioRender.com/opu4lsm, accessed on 10 September 2026).

Historically, chemotherapy-associated cardiac injury was often divided into Type I and Type II cardiotoxicity (Figure 1). Type I toxicity was typically associated with anthracyclines and characterized as dose-dependent and potentially irreversible myocardial injury [7], whereas Type II toxicity was typically associated with trastuzumab and considered more likely to be reversible [7]. This distinction was useful for describing broad differences between anthracycline- and trastuzumab-associated cardiac injury. However, this classification is recognized as an oversimplification [6] because the extent, reversibility, and clinical presentation of cardiac dysfunction vary among patients and may depend on treatment exposure, baseline cardiovascular risk, concomitant therapies, and the timing of cardiac assessment [6].

### 3.1. Anthracyclines and Cardiotoxicity

Anthracyclines, including doxorubicin, daunorubicin, epirubicin, and idarubicin, remain important components of treatment for breast cancer, hematological malignancies, and several other solid tumors. However, their clinical utility is limited by cancer therapy-related cardiac dysfunction, which may develop during treatment or become clinically apparent months to years after anthracycline exposure. Although the risk generally increases with cumulative dose, cardiac injury can also occur at lower cumulative exposures, particularly in patients with pre-existing cardiovascular disease, older age, prior thoracic radiotherapy, or concomitant administration of other cardiotoxic therapies [8].

Anthracycline-induced cardiotoxicity is a multifactorial process and should not be regarded solely as a consequence of excessive ROS generation. A central mechanism involves topoisomerase IIβ (Top2β), which is expressed in cardiomyocytes. Doxorubicin stabilizes the Top2β–DNA cleavage complex, resulting in DNA double-strand breaks and altered transcription of genes involved in mitochondrial biogenesis, oxidative phosphorylation, cellular survival, and antioxidant defense. Experimental deletion of Top2β in cardiomyocytes substantially reduces doxorubicin-induced DNA damage, mitochondrial dysfunction, oxidative stress, and heart failure, supporting a major mechanistic role for this pathway [9,10].

Oxidative and nitrosative stress nevertheless remain important components of anthracycline-related cardiac injury. Anthracyclines undergo redox cycling of their quinone moiety, during which semiquinone intermediates transfer electrons to molecular oxygen and generate superoxide and other reactive oxygen species. Doxorubicin also accumulates in mitochondria due to its high affinity for cardiolipin, thereby impairing the electron transport chain, decreasing adenosine triphosphate (ATP) production, disrupting the mitochondrial membrane potential, and promoting the formation of additional reactive oxygen species [10,11]. These effects are particularly relevant in cardiomyocytes because of their high energetic requirements and limited regenerative capacity.

Disruption of iron homeostasis constitutes another important mechanism. Anthracyclines can promote mitochondrial iron accumulation and iron-dependent lipid peroxidation. Experimental evidence has implicated ferroptosis, a regulated form of cell death characterized by iron-dependent phospholipid oxidation and failure of glutathione peroxidase (GPx)-associated antioxidant defenses, in doxorubicin-induced cardiomyopathy. Inhibition of ferroptosis reduces cardiomyocyte death and improves cardiac injury in experimental models, although the relative contribution of ferroptosis compared with apoptosis and other regulated cell-death pathways remains model-dependent [12,13].

Anthracyclines also interfere with mitochondrial quality-control systems. Doxorubicin has been reported to impair lysosomal function, autophagosome processing, and autophagic flux, resulting in the accumulation of damaged proteins and dysfunctional mitochondria. However, the literature regarding autophagy is not entirely uniform, as both excessive autophagic activation and impaired autophagic completion have been described depending on dose, duration, and experimental model. Therefore, dysregulated, rather than uniformly increased or decreased, autophagy appears to be the more accurate interpretation of the current evidence [14].

Additional mechanisms include calcium-handling abnormalities, endoplasmic reticulum stress, sarcomeric disorganization, and activation of apoptosis, necroptosis, feroptosis, and other cell-death pathways. Anthracycline toxicity is also not restricted to cardiomyocytes. Doxorubicin can injure cardiac endothelial cells, impair nitric oxide (NO) -dependent vascular signaling, disrupt microvascular integrity, and promote endothelial apoptosis and dysfunction [15]. Inflammatory signaling, including activation of nuclear factor kappa B (NF-κB) and increased expression of tumor necrosis factor-α (TNF-α) and other cytokines, may further amplify myocardial injury. Persistent inflammation, cellular senescence, extracellular matrix remodeling, and fibrosis may subsequently contribute to progressive ventricular dysfunction.

Taken together, anthracycline cardiotoxicity arises from interacting nuclear, mitochondrial, metabolic, inflammatory, endothelial, and cell-death pathways rather than from a single oxidative mechanism. Top2β-mediated DNA injury and mitochondrial dysfunction are among the most strongly supported mechanisms, whereas the precise contributions of ferroptosis, impaired autophagy, and other regulated cell death pathways remain predominantly defined in preclinical models. This distinction is important when interpreting studies of grape-derived polyphenols because a reduction in oxidative stress biomarkers alone does not establish comprehensive cardioprotection. Future studies should determine whether these compounds also preserve cardiac function, mitochondrial quality control, iron homeostasis, and antitumor efficacy.

### 3.2. Trastuzumab and Cardiotoxicity

Trastuzumab is a humanized monoclonal antibody directed against HER2/ErbB2, which is overexpressed or amplified in approximately 15–20% of breast cancers. The introduction of trastuzumab into neoadjuvant, adjuvant, and metastatic treatment has substantially improved oncological outcomes. Nevertheless, HER2-targeted therapy is associated with cancer therapy-related cardiac dysfunction, most frequently presenting as an asymptomatic reduction in left ventricular ejection fraction and, less commonly, symptomatic heart failure [8,16].

The myocardial effects of trastuzumab are mechanistically distinct from, although clinically interconnected with, anthracycline-induced injury. In the adult heart, ErbB2 forms functional heterodimers, particularly with ErbB4, in response to neuregulin-1 released mainly by cardiac endothelial cells. Neuregulin-1/ErbB4/ErbB2 signaling supports cardiomyocyte survival, mitochondrial integrity, calcium handling, sarcomeric organization, and adaptation to hemodynamic, metabolic, and oxidative stress. Cardiac-specific deletion of ErbB2 in experimental models results in dilated cardiomyopathy, demonstrating that intact ErbB2 signaling is necessary for normal myocardial function [17].

Inhibition of ErbB2 signaling can directly impair cardiomyocyte energetics. Experimental blockade of ErbB2 has been shown to alter the balance of pro- and anti-apoptotic Bcl-2 family proteins, reduce mitochondrial membrane potential, decrease intracellular ATP concentrations, and activate mitochondrial apoptotic signaling [11]. In human induced pluripotent stem cell-derived cardiomyocytes, clinically relevant trastuzumab exposure impaired contractility and calcium handling and produced abnormalities in mitochondrial energy metabolism without causing the same degree of direct cell death or sarcomeric disorganization observed with doxorubicin [18]. These findings support a predominantly functional and metabolic phenotype but do not exclude structural injury in susceptible patients or after prolonged exposure.

Trastuzumab has additionally been reported to dysregulate basal autophagy and increase ROS production in human cardiomyocytes. Nevertheless, these findings are mainly derived from cellular models, and the relative contribution of autophagy, oxidative stress, mitochondrial dysfunction, and inflammatory signaling in patients remains incompletely defined [19]. Thus, trastuzumab-associated cardiac dysfunction should not be reduced to a single pathway.

The traditional distinction between irreversible anthracycline-related “type I” cardiotoxicity and reversible trastuzumab-related “type II” cardiotoxicity is now considered overly simplistic. Left ventricular function frequently improves after interruption of HER2-targeted therapy and initiation of appropriate cardiovascular treatment, but recovery is not universal. In a clinical cohort of patients with cancer therapy-associated left ventricular dysfunction, most patients improved following cardiovascular intervention, although a proportion did not achieve complete recovery [20]. Current cardio-oncology guidance therefore uses the broader concept of cancer therapy-related cardiovascular toxicity rather than relying exclusively on the historical type I/type II classification [8].

Previous or concurrent anthracycline exposure substantially increases susceptibility to trastuzumab-associated cardiac dysfunction. Anthracyclines may produce subclinical DNA, mitochondrial, endothelial, and structural injury, whereas ErbB2 signaling contributes to myocardial stress adaptation and repair. Subsequent HER2 blockade can therefore reduce cardiomyocytes’ ability to compensate for or recover from prior anthracycline damage. Experimental evidence also indicates that trastuzumab exposure may reduce cardiomyocyte Top2β expression and modify the cellular response to anthracyclines, providing a possible molecular basis for the enhanced cardiac risk observed with combined or sequential treatment [21].

Established clinical risk factors include prior anthracycline exposure, older age, hypertension, pre-existing cardiovascular disease, and reduced or borderline baseline left ventricular function. Baseline cardiovascular risk assessment and serial cardiac surveillance are therefore recommended during HER2-targeted therapy. Echocardiography, including left ventricular ejection fraction and, where available, global longitudinal strain, is commonly used, while cardiac biomarkers may provide complementary information in selected patients [8].

Direct evidence supporting the use of grape-derived polyphenols specifically for the prevention of trastuzumab-associated cardiotoxicity remains scarce. Most experimental studies evaluating resveratrol, proanthocyanidins, quercetin, catechins, or grape-derived extracts have used doxorubicin or other anthracycline-based models. Although oxidative stress, mitochondrial dysfunction, inflammation, and impaired autophagy may overlap in anthracycline- and trastuzumab-induced injury, findings from doxorubicin models cannot be automatically extrapolated to HER2-targeted therapy because the initiating molecular mechanisms differ.

The potential cardioprotective role of grape-derived polyphenols during trastuzumab treatment remains mechanistically plausible but insufficiently demonstrated. Dedicated studies using trastuzumab-exposed human cardiomyocytes, appropriate animal models, and patients receiving HER2-targeted therapy are needed. Such investigations should evaluate functional cardiac endpoints, clinically relevant biomarkers, achievable circulating concentrations of polyphenols and their metabolites, and possible effects on HER2-targeted anticancer efficacy before routine supportive use can be considered.

### 3.3. Biomarkers of Cardiotoxicity

Early detection of cancer therapy-related cardiotoxicity is important because subclinical myocardial injury may precede overt functional impairment. Current cardio-oncology guidelines support an integrated approach combining circulating cardiac biomarkers with cardiovascular imaging rather than relying on a single marker. Cardiac troponin (cTnI or cTnT) and natriuretic peptides (BNP or NT-proBNP) are the most established circulating biomarkers for monitoring myocardial injury and hemodynamic stress, respectively, whereas emerging markers of oxidative stress and inflammation, including myeloperoxidase (MPO) and high-sensitivity C-reactive protein (hs-CRP), remain investigational [8].

Cardiac troponins provide a sensitive measure of myocardial injury during potentially cardiotoxic cancer therapy and have been investigated as early predictors of subsequent ventricular dysfunction. In a prospective study of 204 patients with aggressive malignancies receiving high-dose chemotherapy, Cardinale et al. reported an increase in circulating cTnI in 65 patients (32%). During seven months of echocardiographic follow-up, cTnI-positive patients experienced a more pronounced and persistent reduction in left ventricular ejection fraction (LVEF) than cTnI-negative patients, and the magnitude of cTnI elevation was strongly correlated with the subsequent maximum decline in LVEF (r = −0.87, *p* < 0.0001) [22]. These findings support serial troponin assessment as a marker of early myocardial injury; however, elevations should be interpreted in the context of baseline values, treatment exposure, comorbidities, and concomitant cardiac imaging rather than as an isolated measure of cardiotoxicity.

BNP and NT-proBNP provide complementary information by reflecting myocardial wall stress rather than direct cardiomyocyte injury. Changes in natriuretic peptide concentrations have been associated with alterations in cardiac function during anthracycline- and HER2-targeted therapy, although their predictive performance has varied across studies [23]. Consequently, current cardio-oncology guidance recommends baseline measurement when natriuretic peptides are intended for longitudinal surveillance and emphasizes interpretation of serial changes in conjunction with clinical and imaging findings [8].

MPO and hs-CRP may be particularly relevant as exploratory biomarkers in studies of polyphenols because they reflect biological pathways potentially targeted by these compounds, including oxidative stress and inflammation. In a prospective cohort of patients with breast cancer receiving doxorubicin, taxanes, and trastuzumab, Ky et al. found that increases in both troponin I and MPO were associated with subsequent cardiotoxicity, supporting the potential value of a multimarker approach [24]. Nevertheless, evidence for routine clinical use of MPO remains insufficient, and it should currently be considered a mechanistic or exploratory rather than an established cardiac biomarker.

Similarly, hs-CRP has shown potential but limited specificity. In a prospective pilot study of women with HER2-positive early-stage breast cancer receiving trastuzumab, hs-CRP ≥3 mg/L predicted a clinically significant decline in LVEF with a sensitivity of 92.9% but a specificity of only 45.7%; the negative predictive value was 94.1% [25]. Although the high sensitivity and negative predictive value are potentially informative, the low specificity substantially limits the usefulness of hs-CRP as a stand-alone predictor of cardiotoxicity. hs-CRP should therefore be regarded as a complementary or exploratory inflammatory biomarker rather than an independent clinical decision-making tool.

For future clinical studies evaluating the cardioprotective effects of polyphenols during cancer therapy, a standardized multimodal assessment incorporating serial cTnI/cTnT and BNP/NT-proBNP measurements together with echocardiographic assessment of LVEF and global longitudinal strain (GLS) would provide clinically and mechanistically complementary endpoints. GLS is particularly relevant because changes in myocardial deformation may identify subclinical systolic dysfunction despite preserved LVEF, and relative decline in GLS of >15% from baseline is incorporated into contemporary definitions of mild asymptomatic CTRCD [8]. MPO and hs-CRP, together with other validated measures of oxidative stress and inflammation where appropriate, may be incorporated as secondary mechanistic endpoints. Such an approach would allow future polyphenol trials to determine not only whether an intervention modifies oxidative or inflammatory pathways, but also whether these biological effects translate into preservation of myocardial function and clinically meaningful attenuation of cancer therapy-related cardiotoxicity.

## 4. Grape Polyphenols and Their Biological Activities

Polyphenols, a group of natural compounds with antioxidant and anti-inflammatory properties, have shown considerable potential for protecting against cancer therapy-induced cardiac damage. These compounds have been found to reduce oxidative stress, necrosis, and apoptosis in the heart, thereby preserving cardiac function [26]. Among them, grape-derived polyphenols have attracted particular attention due to their rich composition and well-documented biological activity (Table 1).

Numerous experimental studies have demonstrated the antioxidant activity of dietary polyphenols, suggesting that it may contribute to their cardioprotective properties [27,28,29]. Earlier research has suggested that the use of polyphenols may represent a potential strategy for protecting the cardiovascular system from the effects of chemotherapeutic agents. For instance, our earlier findings showed that a grape polyphenol concentrate significantly attenuated doxorubicin-induced cardiotoxicity in adult Wistar rats [30]. Animals that received polyphenol treatment had decreased levels of free radicals and increased antioxidant enzyme activity. Aspartate aminotransferase (AST) was also reduced [30]. However, further studies in humans are needed in this area [31].

Polyphenols have also been shown to enhance the therapeutic efficacy of chemotherapy in preclinical studies [32,33,34]. Importantly, these compounds may simultaneously exert cardioprotective effects [35]. A 2024 systematic review of strategies for reducing cardiotoxicity during chemotherapy highlighted that non-pharmacological interventions with antioxidant properties, such as resveratrol (a key grape-associated polyphenol) or curcumin, showed promising results in reducing anthracycline-induced cardiotoxicity and may be useful as adjunctive therapy during cancer treatment [36]. A summary of selected preclinical studies investigating the cardioprotective effects of grape-derived products and grape-associated polyphenols is presented in Table 1 and Table 2.

**Table 1:** Experimental studies evaluating the cardioprotective activity of grape-derived polyphenols in vitro and in vivo.

No.	Type of Polyphenol	Source and Chemical Characterization	Major Constituents (Quantitative Composition)	Study Design	Cardiac Endpoints	Molecular Endpoints	Overall Cardioprotective Effect	Limitations	Type of Study, Source
1	Grape polyphenol concentrate	Source: seed ridges and grape skin of Cabernet Sauvignon Water–alcohol feedstock extraction was used to obtain polyphenol concentrate, followed by evaporation on a rotary evaporator.	Quantitative analysis was not performed, total concentration of phenolic derivatives was 10,000 mg/L.	Species: Wistar rats Weight: 200 ± 20 g *n*:12 Sex: not reported Groups: 3 (DOX *n* = 7, polyphenols + DOX *n* = 9, control *n* = 6) Polyphenol dose: 25 mg/kg/day 7 days Route: Intragastric (i.g.) DOX dose: 8.0 mg/kg, single dose after one week adaptation period Route: Intraperitoneal (i.p.) Control: 0.5 mL drinking water (healthy animals).	Grape polyphenol concentrate partially restored endothelial and myofibrillar ultrastructure. In addition, polyphenol treatment reduced mitochondrial damage and cardiomyocyte edema. However, serum cTnT levels were not significantly normalized by grape polyphenol treatment.	Treatment with grape polyphenols enhanced SOD, CAT, and GPx activities, attenuated free radical/ROS generation compared to doxorubicin group, and reduced AST.	Grape polyphenols concentrate attenuated DOX-induced myocardial injury and oxidative cardiotoxicity, with partial restoration of myocardial ultrastructure and antioxidant defense.	Non-tumor-bearing animals; therefore, antitumor efficacy was not assessed; individual polyphenols were not quantitatively characterized; cardiac function was not directly evaluated; small sample size; only a single dose was tested; no group which would receive just polyphenol without DOX treatment.	In vivo [30]
2	Grape seed extract (GSE)	Commercially obtained (Shaanxi Jintai Biological Engineering Co., Ltd. (Xi’an, China) (CAS No.: 84929-27-1))	Identification and quantification of polyphenols were performed using HPLC method. Ten compounds were identified: gallic acid (1623.68 µg/g), chlorogenic acid (2925.33 µg/g), caffeic acid (1990.29 µg/g), syringic acid (121.76 µg/g), Coumaric acid (342.14 µg/g), Pyro Catechol (719.14 µg/g), Quercetin (110.31 µg/g), Catechin (11,385.07 µg/g), Methyl Gallate (1692.11 µg/g), Apigenin (12.91 µg/g).	Species: Albino rats Weight: 300–400 g Sex: male *n* = 60 Groups: 6 (*n* = 10/group *; control, GSE, DOX, GSE + DOX) Polyphenol dose: 100 mg/kg/day for 35 days. Route: i.g. DOX dose: 10 mg/kg on day 28 Route: i.p. Control: distilled water, i.p. saline on day 28 (healthy animals).	GSE ameliorated DOX-induced ECG abnormalities; reduced myocardial edema, inflammation and myofibrillar damage, and significantly decreased serum cardiac injury markers, including creatine kinase-MB (CK-MB), lactate dehydrogenase (LDH), AST, cTnI, and NT-proBNP compared to DOX group.	GSE reduced lipid peroxidation, oxidative stress, and inflammatory biomarkers (interleukin-1 beta (IL-1β), TNF-α, MPO, and NF-κB) compared with the DOX group, while enhancing antioxidant enzyme activity.	GSE attenuated DOX-induced cardiotoxicity by improving cardiac injury markers and ECG abnormalities, reducing myocardial structural damage, and mitigating oxidative and inflammatory stress. However, L-carnitine was also evaluated and demonstrated a stronger cardioprotective effect than GSE.	Non-tumor-bearing animals; therefore, antitumor efficacy was not assessed; only a single dose was tested; use of a complex extract limits attribution of cardioprotective effects to individual constituents.	In vivo [37]
3	Grape seed (GS) extract	Source: Grape seeds	Quantitative analysis was not performed	Species: Sprague–Dawley rats Weight: 300–400 Sex: male *n* = 28 Groups: 4 (*n* = 7/group; control, DOX, GS, GS + DOX) Polyphenol dose: 100 mg/kg/day for 35 days Route: i.g. (orogastric tube) DOX dose: 10 mg/kg, single dose on day 28 Route: i.p. Control: standard nutrition (healthy animals).	GSE treatment decreased cardiotoxicity biomarkers, including troponin and NT-proBNP, and alleviated histopathological alterations characterized by myocyte misalignment, myocardial hypertrophy, small vessel damage, and fibrosis.	GSE attenuated oxidative stress caused by doxorubicin, as evidenced by decreased levels of malondialdehyde (MDA) and total oxidant status (TOS).	Grapeseed extract reduced doxorubicin-induced cardiotoxicity, as evidenced by lower troponin and NT-proBNP levels, reduced oxidative stress (MDA and TOS), increased antioxidant capacity (TAC and NO), and attenuated histopathological cardiac injury, including myocyte misalignment, hypertrophy, small-vessel disease, and fibrosis.	Non-tumor-bearing animals; therefore, antitumor efficacy was not assessed; only a single dose was tested; no quantitative analysis of grape seed extract; small sample size; additionally, authors reported that doxorubicin was administered intraperitoneally which could possibly lead to peritonitis-related cardiac injury.	In vivo [38]
4	Grape seed (GSE) extract	Source: Grape seeds (*Vitis vinifera*)	Quantitative analysis was not performed	Species: Wistar rats Weight: 180–220 g; Sex: male *n* = 18 Groups: 3 (*n* = 6/group; control, DOX, GSE + DOX) Polyphenol dose: 100 mg/kg/day for 16 days Route: i.p. DOX dose: 2 mg/kg/48 h for 12 days. Note: In the GSE + DOX group, DOX administration started on day 4 at the same dose and frequency Route: i.p. Control: saline i.p. (healthy animals).	EF and fractional shortening (FS) were restored by GSE, while LV contractility, LV pressures, end-diastolic pressure, maximum/minimum dP/dt, heart rate, RR interval, heart weight/body weight ratio, myocardial histopathology, and inflammatory-cell infiltration were significantly improved compared with DOX. LV dimensions, QT interval, and arterial blood pressure were not significantly affected.	Not assessed	Marked cardioprotective effect against DOX-induced cardiac dysfunction and myocardial injury, with improvement of ventricular function, selected ECG and hemodynamic parameters, and attenuation of histopathological and inflammatory changes.	Small sample size; only a single dose was tested; no direct mechanistic or biochemical assessment; individual GSE polyphenols not quantitatively characterized; non-tumor-bearing animals; however, authors noted that in vitro cytotoxicity assessment showed no negative effect of GSE on DOX toxicity in MCF-7 cancer cell line; no group which would receive just GSE without DOX treatment.	In vivo [39]
5	Grape Seed and Skin Extract (GSE)	Source: Grape cultivar (Carignan) of *Vitis vinifera*	Nor assessed	Species: Wistar rats Weight: 220–240 g; Sex: female *n* = 24 Groups: 4 (*n* = 6/group; control, DOX, DOX + GSE, GSE) Polyphenol dose: 500 mg/kg/day for 8 days Route: i.p. DOX dose: 20 mg/kg on day 4 Route: i.p. Control: ethanol ** 10% i.p. (healthy animals) I/R protocol: After treatment, hearts were rapidly isolated and perfused ex vivo using a Langendorff system. Following 10 min stabilization, hearts were subjected to 45 min ischemia followed by 10 min reperfusion.	Treatment with GSE improved heart rate and developed pressure after ischemia/reperfusion (I/R) injury. Additionally, GSE administration partially restored myocardial architecture and attenuated Dox-induced fiber swelling and perinuclear vacuolization.	GSE treatment mitigated lipoperoxidation and carbonylation and normalized the activities of CAT and peroxidase (POD). In addition, total SOD activity was enhanced. GSE also attenuated the Dox-induced increase in myocardial H_2_O_2_ and free iron levels.	GSE exerted a cardioprotective effect by improving cardiac function, partially restoring myocardial architecture, and attenuating Dox-induced oxidative stress.	Non-tumor-bearing animals; therefore, antitumor efficacy was not assessed; only a single dose was tested; small sample size; individual polyphenols were not quantitatively characterized.	In vivo [40]
6	Catechin	Pure compound (Sigma Chemical Co., St. Louis, MO, USA)	-	Species: Wistar rats weight: 200–230 g Sex: male *n* = 45 Groups: 3 (*n* = 15/group; control, adriamycin (ADR), ADR + catechin) Polyphenol dose: 10 mg/kg/day for 4 weeks (2 weeks before and 2 weeks during adriamycin treatment) Route: i.g. Adriamycin dose: 13 mg/kg cumulative dose administered as 6 injections over 2 weeks Route: i.p. Control: distilled water (healthy animals).	Catechin treatment reduced myocardial tissue damage, with fewer myocyte rupture foci and less inflammatory infiltration. In addition, catechin improved myocardial histopathology and decreased serum levels of cTnI.	Activity of CAT, GPx and SOD was significantly increased in comparison with group that received only adriamycin. TNF-α and NF-κB expression were downregulated, whereas iNOS expression was not significantly altered.	Reduced cardiac injury, oxidative stress, and inflammation; restored antioxidant defenses and improved myocardial histopathology.	Non-tumor-bearing animals; therefore, antitumor efficacy was not assessed; due to absence of catechin inhibitors, it was impossible to confirm the mechanism of action; only a single dose was tested; no groups which would receive just catechin without ADR treatment.	In vivo [41]
7	Proanthocyanidin (Pro)	Pure compound (Huzhou NBC Bio-material CO., LTD., Shanghai, China)	-	Species: male Sprague–Dawley rats; Weight: 180–220 g *n* = 24 Groups: 4 (*n* = 6/group; control, Pro, DOX, Pro + DOX) Polyphenol dose: 70 mg/kg/day for 10 days Route: i.g. DOX dose: 15 mg/kg, single dose on day 7 Route: i.p. Control: normal saline, 1 mL/kg, for 10 days (healthy animals).	Pro administration reduced DOX-induced ECG abnormalities, including heart rate and ST-segment elevation, T wave amplitude, and also reduced susceptibility to arrhythmias.	Reduced myocardial MDA levels and increased GSH levels and SOD activity compared with the DOX group.	Attenuated DOX-induced cardiotoxicity by reducing oxidative injury and improving electrophysiological abnormalities.	Non-tumor-bearing animals; therefore, antitumor efficacy was not assessed; cardiac effects were assessed mainly using ECG and biochemical markers rather than comprehensive measures of cardiac function; only a single dose was tested; small sample size.	In vivo [42]
8	Quercetin (Q)	Pure compound	-	Species: Wistar rats Weight: 150–250 g; Sex: male *n* = 32 Groups: 4 (*n* = 8/group; control, DOX, DOX + Q50, DOX = Q100) Polyphenol dose: Q50–50 mg/kg/day Q100–100 mg/kg/day Route: i.g. DOX dose: 2.5 mg/kg/48 h for 2 weeks Route: i.p. Control: saline i.p. (healthy animals).	Both doses of quercetin showed normalization of NT-proBNP levels. However, administration of Q100 had a more significant effect than Q50. Treatment with Q100 demonstrated significant improvements in LVEF and FS. Q50 and Q100 both restored myocardial ultrastructure; However, treatment with Q50 was less effective, as evidenced by the persistence of some structural damage in cardiomyocytes. Important to note that quercetin treatment (both doses) did not normalize parameters (LVEF and FS) to the levels of the control group.	Quercetin (Q50 and Q100) restored Nrf2 and SOD1 levels toward control values. Additionally, treatment with quercetin tended to reduce DNA damage but the difference was not statistically significant.	Treatment with quercetin helped to attenuate DOX-induced cardiotoxicity by improving cardiac function, antioxidant defense, and myocardial ultrastructure. However, a higher dosage showed a stronger therapeutic effect.	Non-tumor-bearing animals; therefore, antitumor efficacy was not assessed; lack of direct measurement of ROS; incomplete study of the mechanism of Nrf2 activation; no group that would receive just quercetin without DOX treatment; small sample size.	In vivo [43]
9	Catechin and proanthocyanidin B4	Source: grape seed (grape variety and extraction methods are not reported).	-	In vitro study using rat heart-derived H9C2(2-1) cells (ATCC, Manassas, VA, USA), cultured in DMEM supplemented with 10% FBS, penicillin and streptomycin Groups: 4 (Control, DOX, catechin + DOX, proanthocyanidin B4 +DOX) Catechin and proanthocyanidin B4 were tested separately at 100 μM DOX exposure: 20 μM for 24 h.	Reduced DOX-induced morphological injury in H9C2(2-1) cardiomyocytes.	↓ intracellular ROS and apoptosis; ↓, regulated Bax-α and Bcl-2 expression, suppressed apoptotic signaling pathways.	Reduced doxorubicin-induced cardiomyocyte injury.	High polyphenol concentration (100 μM) may limit physiological and translational relevance; the lack of an oncology model, and because of this, no assessment of whether catechin and proanthocyanidin B4 affect the antitumor efficacy of DOX.	In vitro [44]
10	Resveratrol	Pure compound	-	In vitro study using h9c2 (ATCC, CRL-1446, Manassas, VA, USA) model of doxorubicin-induced cardiotoxicity. Groups: 5 (Control, DOX, Dexrazoxane (70 μM) + DOX, Resveratrol (25 μM) + DOX, Carvedilol (25 μM) + DOX Pretreatment duration with cardioprotectants: 3 and 24 h. DOX exposure: 7.045 μM for 24 h after coincubation of cells with cardioprotectants.	3 h pretreatment with cardioprotectants did not demonstrate significant improvement in cell viability. However, results of 24 h pretreatment demonstrated that resveratrol produced the strongest effect.	Assessment of ROS levels demonstrated a significant reduction in ROS production in cells pretreated for 3 h with dexrazoxane and resveratrol, while no changes were observed in cells that received carvedilol; 24 h pretreatment showed that all agents reduced ROS formation, without significant changes between groups.	Resveratrol demonstrated strong overall cardioprotective effect, improving cell survival and reducing DOX-induced ROS production, particularly after 24 h of pretreatment in comparison with dexrazoxane and carvedilol.	The rationale for selecting the 70 μM dexrazoxane concentration was not clearly described; use of cardiomyoblasts instead of cardiomyocytes; limited endpoints; the lack of an oncology model, and because of this, no assessment of whether resveratrol affects the antitumor efficacy of DOX.	In vitro [45]

* only grape-derived polyphenol groups (GSE and GSE + DOX) were considered; L-carnitine groups were excluded. The corresponding data are available in [37]. in the table. ** ethanol is used as a vehicle control because it was also used as a solvent for powdered GSE. ↓, decrease.

**Table 2:** Indirect supporting evidence for the cardioprotective effects of polyphenols in non-chemotherapy-induced cardiovascular injury.

No.	Type of Polyphenol	Source and Chemical Characterization	Major Constituents (Quantitative Composition)	Disease Treated	Study Design	Cardiac Endpoints	Molecular Endpoints	Limitations	Type of Study
1	Resveratrol	Pure compound (CAS 501-36-0, Sigma-Aldrich, St. Louis, MO, USA)	-	I/R injury (ischemia was induced by perfusion with glucose-free Krebs–Henseleit buffer containing 95% N_2_/5% CO_2_, followed by reperfusion with oxygenated buffer (95% O_2_/5% CO_2_)).	Species: C57BL/6 mice Weight: not reported; Sex: male *n* = 30 Groups: 3 (*n* = 10/group; young (4-month-old), aging (22-month-old), aging (22-month-old) + resveratrol) Polyphenol dose: 2.0 mg/kg/day for 6 weeks Route: i.g. Aging and young groups received 20% alcohol (2.0 mg/kg/d) Route: i.g. In addition, male SIRT1 KO mice were used to perform SIRT1 activity assay. However, the conditions are not reported.	Myocardium was protected by resveratrol supplementation in aging mice by improving tolerance to I/R damage.	Resveratrol inhibited apoptosis and upregulated autophagy through the Sirtuin 1 (SIRT1) signaling pathway.	Non-chemotherapy I/R injury model; aged C57BL/6 mouse model limits direct relevance to DOX-induced cardiotoxicity; anticancer efficacy and potential interactions with chemotherapy were not assessed.	In vivo In vivo [46]
2	Quercetin	Pure compound (Cat. no. Q4951, Sigma-Aldrich, St. Louis, MO, USA)	-	β_1_-adrenoceptor autoantibody-induced heart failure	Species: C57BL/6 mice Weight: not reported; Sex: male Groups: 2 (quercetin monotherapy group; quercetin treatment group) Dose: 100 mg/kg/d for 4 weeks after the initial primary immunization Route: i.p.	Quercetin restored myocardial autophagy and improved heart function.	Quercetin activated cardiomyocyte autophagy by promoting MDM2-dependent p53 ubiquitination and degradation.	Quercetin’s ability to regulate autophagy is not fully understood and requires further study.	In vivo, in vitro, bioinformatics [47]
3	White grape pomace (WGP)	Source: Pomace consisted of skins, seeds, and stems, of white wine grapes of *Vitis vinifera* L. Polyphenol extraction was performed with 40% ethanol.	Identification and quantification of polyphenols was performed using HPLC method. Nineteen compounds were identified: flavanols (66.5%), flavonols (8.4%), hydroxybenzoic compounds (7.2%), tannins (17.7%).	Isoproterenol-induced myocardial infarction (myocardial infarction was caused by injection of isoproterenol; 45 mg/kg on day 13 i.p.).	Species: Wistar–Bratislava rats Weight: 200–250 g Sex: male *n* = 50 Groups: 5 (*n* = 10/group; control, myocardial infraction control, WGP1, and WGP2, Lisinopril) Polyphenol dose: WGP1-795 mg/kg/d for 13 days WGP2–397.5 mg/kg/d for 13 days Lisinopril dose–10 mg/kg/d for 13 days Route: i.g. Isoproterenol dose–45 mg/kg on day 13 Route: i.p. Control: both controls received saline i.g.	WGP prevented QT and QTc interval prolongation and reduced tissue damage.	WGP decreased NO and MDA and pro-inflammatory cytokines (TNF-α, Interleukin 6 (IL-6), IL-1β).	Echocardiographic assessment of cardiac function and molecular analyses of proteins involved in inflammation, necrosis, and fibrosis were not performed.	In vivo [48]
4	Proanthocyanidin	Pure compound extracted from *Vitis vinifera,* obtained commercially (Nutra Manufacturing Inc., Greenville, SC, USA).	-	Myocardial ischemic injury	Species: *Rattus norvegicus* rats Weight: 320–340 g Sex: male *n *= 32 Groups: 4 (*n* = 8/group; control, proanthocyanidin group, ischemia group and proanthocyanidin-treated group) Polyphenol dose: 100 mg/kg twice a day for 3 weeks Route: i.g. Control and ischemia groups received standard rat food.	Proanthocyanidin improved myocardial histopathology by reducing ischemic damage and preserving myofibrillar structure.	Administration of proanthocyanidin enhanced antioxidant defense; reduced MDA; increased production of SOD, and CAT; and increased GPx.	Non-chemotherapy ischemia model; small sample size; animal study with no assessment of tumor response or chemotherapy efficacy; optimal dose, duration and timing were not established; cardiac protection was assessed mainly by histological and biochemical endpoints, with limited functional assessment.	In vivo [49]
5	Grape seed proanthocyanidin extract (GSPE)	Source: Natural water–alcohol extract from red grape seeds; commercial IH636 grape seed proanthocyanidin extract (ActiVin, InterHealth Nutraceuticals, Benicia, CA, USA).	~54% dimeric proanthocyanidins, 13% trimeric proanthocyanidins and 7% tetrameric oligomeric proanthocyanidins; small amounts of monomeric flavonoids and high-molecular-weight oligomeric proanthocyanidins.	I/R-induced myocardial injury (Ex vivo global ischemia induced by clamping the left atrial inflow and aortic outflow lines for 30 min, followed by 2 h reperfusion).	Species: Sprague–Dawley rats Weight: 320–340 g Sex: not reported *n* = 36 Groups: 3 (*n* = 12/group; control, proanthocyanidin group 50, proanthocyanidin group 100) Polyphenol dose: 50 mg/kg/day for 3 weeks 100 mg/kg/day for 3 weeks Route: i.g. Control: methylcellulose–saline solution.	GSPE reduced reperfusion-induced ventricular fibrillation (VF) and ventricular tachycardia (VT) and improved postischemic recovery of coronary flow, aortic flow, and left ventricular developed pressure (LVDP). At 100 mg/kg, recovery of coronary flow, aortic flow, and developed pressure was improved by 32% ± 8%, 98% ± 8%, and 37% ± 3%, respectively.	Myocardial oxygen free-radical formation measured by electron spin resonance (ESR). GSPE significantly inhibited free-radical formation; at 100 mg/kg, free-radical intensity was reduced by 75% ± 7%.	Animal/isolated-heart study; non-chemotherapy ischemia model; animal study with no assessment of tumor response or chemotherapy efficacy; cardioprotection was evaluated primarily through postischemic functional recovery, arrhythmias and free-radical formation.	In vivo [50]

### 4.1. Classification of Grape Polyphenols

**Figure 2:**
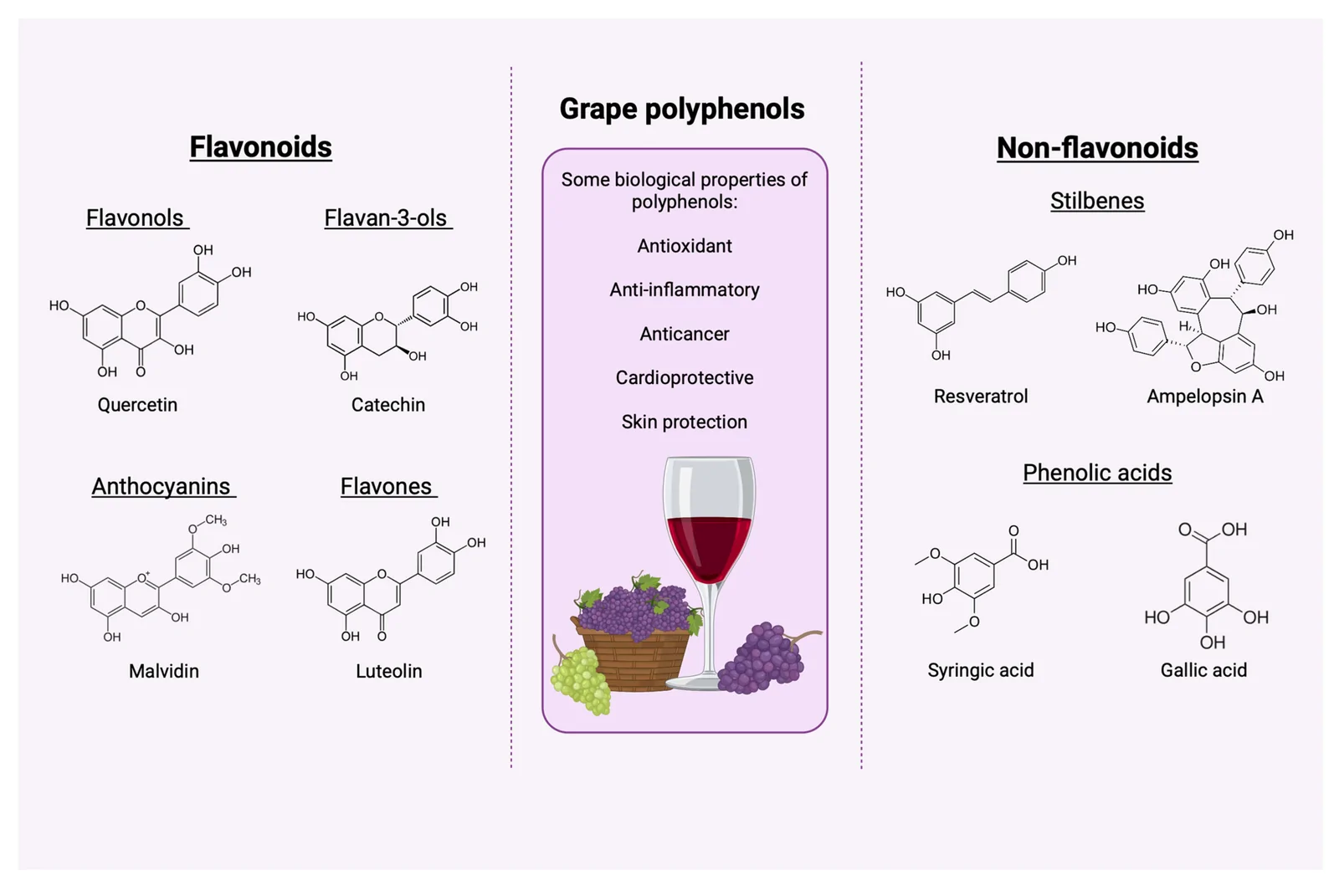
Common polyphenols found in grapes (Created in BioRender. Maikenova, A. (2026) https://BioRender.com/jc70jre, accessed on 9 September 2026).

Polyphenols are a class of chemical compounds characterized by the presence of more than one phenolic group in their molecular structure. They have diverse structures and properties. Therefore, polyphenols are divided into different categories. For example, they can be divided into flavonoid compounds and non-flavonoid compounds, as shown in Figure 2 [51]. Grapes represent a rich source of both types of polyphenols, with a very diverse composition, including well-known compounds such as resveratrol, quercetin, catechins, anthocyanins, and gallic acid, as well as many other beneficial bioactive compounds. According to Hu et al., 53 types of polyphenols were identified in grapes and grape-derived products, including 33 anthocyanins, 12 flavonols, 4 phenolic acids, and 4 flavanols/proanthocyanidins [52]. However, the composition and concentration of polyphenols vary depending on grape variety, cultivation conditions, and processing methods, which may influence their biological activity.

Importantly, grape-derived polyphenols have been widely studied for their cardioprotective properties, particularly in the context of oxidative stress and cardiovascular damage. Although most of the available evidence has been obtained from experimental models and requires cautious extrapolation to humans, these findings support further investigation of grape-derived polyphenols as potential adjunctive cardioprotective agents in oncology. However, their clinical applicability, including appropriate dosing, safety, and potential interactions with anticancer therapies, remains to be established.

#### 4.1.1. Non-Flavonoids

Stilbenes are a group of non-flavonoid phenolic compounds found in several plant species, with grapes being one of the most important dietary sources [53]. These compounds are synthesized by plants in response to biotic or abiotic stress. Stilbenes have a wide range of biological activities beneficial to human health [53]. It is also believed that stilbenes are grape phytoalexins present in berries and are responsible for the beneficial effects of wine consumption. The predominant stilbene identified in grapes is resveratrol, known for its anti-inflammatory, anticancer, antioxidant, and cardioprotective activities [54]. This substance can inhibit platelet aggregation. The properties of resveratrol have been repeatedly noted in in vivo and in vitro studies. In an in vivo study by Ibrahim et al., resveratrol supplementation improved the levels of glutathione (GSH), glutathione reductase (GR), SOD, CAT, and acetylcholinesterase (AChE) [55]. According to Xia et al., resveratrol actively scavenges various oxidants, including the hydroxyl radical, superoxide, and hydrogen peroxide [56].

Phenolic acids are a subtype of non-flavonoid polyphenols that are further classified into hydroxybenzoic (C6-C1) and hydroxycinnamic acids (C6-C3), including their derivatives [57]. Typically, phenolic acids exist in nature as conjugated forms in plants and foods and are seldom found in free forms [57]. Major hydroxycinnamic acids found in grape pomace are p-coumaric, caffeic, and ferulic acids [58]. They possess strong antioxidant, antimicrobial, and anti-inflammatory properties. Some data suggest that they may also have anticancer effects [59]. For instance, major acids such as caffeic and ferulic acids have been shown to exhibit anticancer activity in liver cancer [60] and breast cancer [61]. The major hydroxybenzoic acids found in grapes include gallic acid (GA), ellagic acid, protocatechuic acid, vanillic acid, and syringic acid [57]. Like hydroxycinnamic acids, hydroxybenzoic acids are of great interest as health-enhancing agents due to their antiviral, antioxidant, antimicrobial, and anticancer effects [62].

The total content of phenolic acids (both hydroxycinnamic and hydroxybenzoic acids) in grapes varies depending on cultivar, geographical origin [57], genetic factors, climatic conditions, ripening stage, and extraction method [63]. For instance, Nicolescu et al. performed a comparison of three grape varieties: Merlot, Pinot Noir, and Feteasca Neagra. The results showed that the highest concentration of phenolic acids in grape seeds was observed in Pinot Noir (169.53 ± 7.32 fmg GA-eq/g) [63].

#### 4.1.2. Flavonoids

Flavonols are secondary metabolites found in grape berries and other higher plants. The most well-known flavonols present in grape berries are isorhamnetin, kaempferol, laricitrin, myricetin, quercetin, syringetin, and taxifolin [64]. They are typically found in grape skin as 3-O-glycosides and sometimes as 7-O-glycosides or aglycones [64]. Flavonols are responsible for copigmentation together with anthocyanins and are used as markers in grape taxonomy [54]. Additionally, this class of polyphenols also exhibits numerous other biological activities, such as anticancer, antidiabetic, anti-inflammatory, antimalarial, antioxidant, antiviral, cardioprotective, hepatoprotective, and neuroprotective activities [65]. For example, one of the most extensively studied polyphenols, quercetin, has attracted considerable scientific interest for many years due to its pronounced biological activity. Dulf et al. reported that oral administration of quercetin reduced biomarkers associated with doxorubicin-induced cardiotoxicity in Wistar rats [43]. Histopathologic analysis showed a significant difference between animals receiving only doxorubicin and those that were administered quercetin. The percentage of degenerative myocardial cells decreased, as did the number of inflammatory cells and the area of fatty infiltration and blood stasis in blood vessels in the quercetin-treated groups [43].

Anthocyanins are a water-soluble subclass of flavonoids and are also natural pigments responsible for red, purple, and blue coloration in plants [58,66]. They are a glycosylated form of anthocyanidins (aglycones) [66]. At present, more than 600 anthocyanin compounds have been identified in plants. These compounds can be categorized into six major aglycone forms based on their substituent patterns: cyanidin (Cy), delphinidin (Dp), malvidin (Mv), pelargonidin (Pg), peonidin (Pn), and petunidin (Pt) [67]. Anthocyanidins usually exist in nature in the form of anthocyanins due to their unstable chemical form, and glycosylation helps to form anthocyanins with enhanced molecular stability and improved water solubility [68]. Like other polyphenols, anthocyanins are bioactive compounds with a broad spectrum of applications. For instance, dietary supplementation with anthocyanins has been shown to reduce inflammation by inhibiting signaling pathways, such as NF-κB [69,70]. However, their therapeutic potential is very limited due to their extremely low bioavailability; only 1–2% of the administered compound remains in its original form after administration [71]. The positive effect of anthocyanins can be enhanced by increased intake of anthocyanin-rich foods, but there are no reports on the side effects of excessive consumption of such products [72]. Therefore, another strategy for overcoming the issue of low bioavailability is encapsulation and delivery of anthocyanins via different drug-delivery platforms such as exosomes, phytosomes, liposomes, etc. [70].

Flavan-3-ols or flavanols are one of the most common phytochemicals present in grapes. They can be found both in the skin and the seeds of grapes [58]. They are a subclass of flavonoids characterized by the presence of the hydroxyl group (-OH) at the C3 position of the C ring [58]. Catechin and epicatechin are classified as flavanols, and they can further polymerize to form oligomers and polymers such as proanthocyanidins (also known as condensed tannins) [58]. Flavan-3-ols can also be esterified with gallic acid, resulting in the formation of new bioactive compounds, including catechin gallate, epicatechin gallate, epigallocatechin, epigallocatechin gallate, and gallocatechin [58]. Their presence in food is responsible for aroma, acidity, astringency, bitterness, sweetness, and viscosity of saliva. Flavan-3-ols have also been proven to improve the microbial and oxidative stability of food [73].

### 4.2. Biological Properties of Polyphenols and Their Therapeutic Applications

#### 4.2.1. Antioxidant Activity

Polyphenols represent a class of powerful antioxidant agents. This activity is mediated through four main mechanisms: scavenging free radicals, metal chelation, an increase in the number of endogenous enzymes that synthesize antioxidants, and inhibition of ROS-forming enzymes [74]. Direct scavenging of ROS is associated with the structural features of polyphenols, which allow them to donate electrons or hydrogen atoms [75]. Highly reactive ROS such as hydroxyl radicals or superoxide anions transform into less reactive and stable phenoxyl radicals, which are less harmful [76]. Another mechanism that successfully decreases levels of ROS is metal chelation. Polyphenols act as metal ion chelators by using their hydroxyl groups to bind transition metals such as iron (Fe), copper (Cu), or zinc (Zn), leading to the formation of metal–polyphenol complexes, thereby preventing the Fenton reaction and inhibiting ROS production [77]. As a third mechanism, some polyphenols can activate endogenous defense systems by upregulating enzymes such as GPx, SOD, and CAT via the Nrf2/ARE signaling pathway to reduce oxidative stress [78,79]. For instance, this ability was demonstrated for resveratrol in a study by Xu et al. focusing on myocardial I/R injury in diabetic rats. Resveratrol reduced oxidative stress and myocardial damage in experimental rats by activating the AMPK/p38/Nrf2 pathway, which resulted in upregulation of Nrf2 downstream antioxidant enzymes and increased levels of endogenous antioxidants [80]. Moreover, polyphenols may enhance the expression of antioxidant and phase II detoxifying enzymes (e.g., glutathione S-transferase (GST), heme-oxygenase-1 (HO-1)), contributing to the maintenance of redox homeostasis and protection of cells from ROS-mediated damage [78]. Lastly, polyphenols can downregulate enzymes involved in ROS production, such as lipoxygenase (LOX) and cyclooxygenase (COX). This ability enables polyphenols to reduce the production of key inflammatory mediators, including prostaglandins, leukotrienes, arachidonic acid, and NO [81].

#### 4.2.2. Anticancer Activity

Polyphenols have been found to exhibit promising anticarcinogenic effects. They can affect cancer cells through different mechanisms, which include disrupting signal transduction pathways, inhibiting cell cycle events, inducing apoptosis, suppressing tumor cell proliferation, and inhibiting angiogenesis [82,83]. Angiogenesis is the process of new blood vessel formation from pre-existing vasculature and is involved in several physiological processes, including embryogenesis, wound healing, and all stages of the menstrual cycle [84]. However, in cancer, angiogenesis leads to the formation of new blood vessels that provide a supply of oxygen and nutrients for cancer cells to sustain their growth [85]. Additionally, new tumor blood vessels are abnormal and leaky; they form a chaotic blood flow pattern with high permeability, facilitating the entry of cancer cells into the bloodstream and subsequent metastasis [86]. Polyphenols can exhibit antiangiogenic activity. This effect has been observed in multiple studies. Early reports of the antiangiogenic activity of polyphenols date back to the 2000s [87]. Similarly, grape stem extract has been shown to inhibit angiogenesis in human endothelial cells (EA.hy926) via suppression of tube formation. The effect was attributed to inhibition of vascular endothelial growth factor (VEGF), which was accompanied by apoptosis in EA.hy926 cells. Analysis of hypoxia-inducible factor 1-alpha (HIF-1α) and cyclooxygenase (COX-1) levels showed no significant changes in their levels, indicating that suppression of VEGF occurs in an HIF-1α- and COX-1-independent manner [88].

Polyphenols can upregulate PI3K/Akt protein expression, activate the expression of p53 and CaMKII, and reduce ROS production and phosphorylation, inducing cell apoptosis [89]. These processes lead to cell cycle arrest (commonly in G1 phase), thereby inhibiting the proliferation of malignant cells. Moreover, polyphenols can suppress metastasis by modulating proteins responsible for epithelial–mesenchymal transition (EMT). Therefore, cells cannot change their epithelial morphology into a mesenchymal one, thereby limiting their migration and invasion into surrounding tissues [89]. In 2021, Yang et al. provided further evidence supporting this concept [90]. They demonstrated that grape seed proanthocyanidins (GSPs) inhibited the migration and invasion of bladder cancer cells by reversing transforming growth factor-β (TGF-β)-induced epithelial–mesenchymal transition (EMT). GSP treatment shifted the balance toward an epithelial phenotype by upregulating the epithelial markers E-cadherin and ZO-1, while downregulating the mesenchymal markers N-cadherin, vimentin, and Slug. In addition, GSPs suppressed the TGF-β signaling pathway, further contributing to the inhibition of EMT and tumor cell invasiveness [90].

In addition to suppressing angiogenesis and epithelial–mesenchymal transition, grape-derived polyphenols can directly inhibit cancer cell survival by inducing apoptosis. In 2021, Quero et al. demonstrated that grape stem extract exhibited potent antioxidant and anticancer activities [91]. Phenolic profiling identified nine major compounds, with (+)-catechin and a quercetin-3-derivative among the predominant constituents. In vitro experiments showed that treatment with grape stem polyphenolic extracts inhibited cancer cell growth by inducing apoptosis through alterations in intracellular ROS levels and mitochondrial membrane potential. The increase in ROS levels was partly attributed to the inhibition of the antioxidant enzyme thioredoxin reductase 1 (TrxR1), while suppression of NF-κB signaling contributed to the induction of apoptosis [91]. In another study, Kita et al. demonstrated that piceatannol inhibited the proliferation and invasion of AH109A hepatoma cells in both in vitro and in vivo models [92]. Piceatannol exerted a dose-dependent antiproliferative effect: lower concentrations induced cell cycle arrest at the G2/M phase, whereas higher concentrations significantly increased apoptotic cell death. Likewise, piceatannol inhibited hepatocyte growth factor (HGF)-induced signaling in a dose-dependent manner, leading to a marked reduction in the invasive potential of hepatoma cells [92].

Despite the promising anticancer effects described above, there is still a risk that grape-derived polyphenols might not be selective toward malignant cells. An in vitro study by Tsantila et al. investigating extracts from different parts of *Vitis vinifera* showed that most of the obtained polyphenolic extracts did not significantly affect the proliferation of normal MCF-10A breast cells. However, among 12 tested samples extracts E3 and E5 exerted a cytotoxic effect, with IC50 values of 38.49 and 131.8 mg/L, respectively. Interestingly, both extracts had a high polyphenol content (measured in pyrogallol and catechin), but no direct correlation was observed between total phenolic content and cytotoxicity, suggesting that poor selectivity could be associated with specific constituents and other components [93]. Nevertheless, the data available to date predominantly indicates a beneficial antitumor effect of grape polyphenols. However, further studies are required to investigate the safety profiles of individual polyphenols and their specific biological activities that contribute to anticancer effectiveness.

#### 4.2.3. Anti-Inflammatory Activity

Among the many beneficial biological effects of polyphenols, pronounced anti-inflammatory activity has been widely reported [94]. They alleviate inflammation in different ways since the mechanisms of inhibiting the inflammatory response depend on the class of polyphenols [95]. In summary, these pathways include reduction of inflammatory mediators, suppression of key inflammatory enzymes, downregulation of proinflammatory genes, modulation of cell signaling pathways, and attenuation of oxidative stress [94,95]. One of the most extensively studied mechanisms involves the inhibition of NF-κB activation. Polyphenols can regulate the NF-κB pathway in different ways, including inhibition of IκB kinase phosphorylation, modulation of NF-κB binding to DNA, and decreased ROS production [96], leading to a significant reduction in inflammation. For instance, grape-derived polyphenols, such as catechin, quercetin, and resveratrol, have demonstrated the ability to effectively inhibit the NF-κB signaling pathway in in vitro and in vivo studies [97,98,99]. Additionally, the natural ability of polyphenols to act as antioxidant agents plays a crucial role in suppressing inflammatory foci. As described earlier, polyphenols can affect ROS-producing enzymes (COX, LOX, NOS), thereby affecting the expression of major inflammation mediators such as prostaglandins, leukotrienes, and NO [96]. In addition, polyphenols have immunomodulatory activity, which helps reduce inflammation. They regulate the activity and behavior of immune cells, including their migration and recruitment by changing the synthesis of chemokines and adhesion molecules [100]. Furthermore, modulation of immune cell behavior by polyphenols leads to a decrease in the production of inflammatory mediators, significantly enhancing the anti-inflammatory effect of these natural compounds [101].

Several preclinical studies have shown promising anti-inflammatory effects of grape polyphenols [102,103,104]. Effective translation into clinical practice remains uncertain. Results from existing clinical studies remain inconclusive. Deghani et al. in their systematic review and meta-analysis of 29 randomized clinical trials, found that grape and grape-derived products did not significantly reduce inflammatory biomarkers. However, it was noted that none of these trials recruited people with acute inflammation, and baseline levels of inflammatory biomarkers were low. An increase in antioxidant activity was also noted [105]. In another similar study by Haghighatdoost et al. no significant overall effects of grape polyphenol supplementation on IL-6, TNF-α, or hs-CRP were observed [106].

#### 4.2.4. Cardioprotective Activity

Polyphenols have attracted considerable attention due to their potential cardioprotective properties. Earlier, it was believed that the cardioprotective effect was mostly mediated through polyphenols’ ability to scavenge ROS [107]. However, recent studies have emphasized the role of polyphenols in regulating genomic and epigenomic processes [107]. For instance, resveratrol was found to modulate epigenetic histone marks, including H3K27me3. In a study by Han et al., the beneficial effect of resveratrol in treating hypertension induced by deoxycorticosterone acetate (DOCA) salt was associated not only with the modulation of blood biomarkers but also with methylation of the vascular H3K27me3 mark [108]. The experiment was performed on Wistar rats that were administered resveratrol (50 mg/L in drinking water). Later, histology results revealed that staining of H3K27me3 was enhanced in the aorta across all layers in both hypertensive and normotensive animals. In contrast, in the renal artery, resveratrol induced the expression of H3K27me3 only in rats with hypertension, while in normotensive rats, it was diminished [108]. Moreover, resveratrol demonstrated the ability to reduce homocysteine-mediated vascular smooth muscle cell proliferation by reducing PTEN gene methylation. This result suggests that resveratrol may protect blood vessels through an epigenetic pathway [109]. Although these findings were obtained in non-cancer cardiovascular models, they indicate that polyphenols may influence several molecular and vascular pathways that are also involved in cancer therapy-related cardiovascular injury. However, such evidence should be distinguished from direct evidence obtained in chemotherapy-induced cardiotoxicity models.

In addition to gene/epigenetic regulation, polyphenols also protect cardiac function by improving endothelial function, inhibiting platelet aggregation, and modulating lipid metabolism [110]. Polyphenols may modulate platelet activation and aggregation, which could contribute to cardiovascular protection [111]. Phenolic compounds from grape wine and pomace are believed to exert antiplatelet activity. In a study by Rodriguez et al. Merlot wine enriched with *Cabernet Sauvignon* grape pomace phenolic compounds at different concentrations was evaluated for antiplatelet activity, as well as quercetin, epicatechin, catechin, gallic acid, and caffeic acid [112]. The results showed that the fortified wine sample with the highest phenolic content performed best in platelet aggregation and activation tests induced by 3 platelet agonists: TRAP-6, collagen, and adenosine diphosphate, demonstrating a potent ability to inhibit platelet aggregation. Regarding the polyphenols studied individually, quercetin demonstrated the highest antiplatelet activity against all three agonists studied, while gallic acid demonstrated a minimal antiplatelet effect [112]. Although antiplatelet activity may be relevant to broader cardiovascular protection, its direct contribution to chemotherapy-induced cardiac injury remains insufficiently established. Moreover, in oncology patients, the potential antiplatelet effects of grape-derived polyphenols may have both beneficial and clinically relevant adverse implications, particularly in patients receiving concomitant antiplatelet or anticoagulant therapy.

Dietary intake of polyphenols may improve lipid profiles, which is especially important because of the effects of low-density lipoprotein (LDL) oxidation on cardiovascular health. LDL oxidation is a key factor in the development of atherosclerosis and is possibly connected to the increased risk of suffering from myocardial infarction and coronary heart disease [113,114]. However, these mechanisms should not be considered as direct evidence of myocardial protection in patients receiving chemotherapy; effects on lipid metabolism and LDL oxidation may provide indirect support for cardiovascular health.

More direct evidence is available from experimental models of chemotherapy-induced cardiotoxicity. For instance, Razmaraii et al. reported that intraperitoneally administered grape seed extract (GSE) of *Vitis vinifera* helped to mitigate the cardiotoxic effect of doxorubicin treatment, preserving left ventricular function, hemodynamic parameters, and myocardial structure. In addition, an in vitro experiment using MCF-7 breast cancer cells demonstrated that GSE did not have any significant influence on the cytotoxic effect of doxorubicin, suggesting that the cardioprotective activity of GSE can be achieved without compromising the efficacy of anticancer treatment [39]. A similar result was reported by Adiyaman et al. GSE (origin was not reported) demonstrated a cardioprotective effect against doxorubicin-induced cardiotoxicity in Sprague–Dawley rats. Administration of GSE resulted in decreased levels of cardiotoxicity biomarkers, including troponin and NT-proBNP. Additionally, GSE attenuated oxidative stress caused by doxorubicin, as evidenced by decreased levels of MDA and total oxidant status. Histopathological abnormalities were reduced, including myocyte misalignment, myocardial hypertrophy, small vessel damage, and fibrosis. However, the authors reported that doxorubicin was administered intraperitoneally, which could possibly lead to peritonitis-related cardiac injury, which might complicate the interpretation of the obtained results and make it difficult to distinguish doxorubicin-specific cardiotoxicity from injection-associated effects [38]. Nevertheless, these findings provide evidence linking the antioxidant activity of grape-derived polyphenols with functional cardiac outcomes rather than relying solely on biochemical markers.

Further support comes from an in vitro study by Monahan et al., where the authors compared the therapeutic efficacy of resveratrol with known cardioprotective drugs, dexrazoxane and carvedilol, in a model of induced cardiotoxicity in H9c2 cardiomyoblasts. According to the obtained results, none of the tested compounds improved cell survival when they were pre-incubated for 3 h before the addition of doxorubicin. However, after incubation for 24 h, resveratrol showed the most significant result in comparison to other groups. Additionally, assessment of ROS levels demonstrated a significant reduction in ROS production in cells pre-treated for 3 h with dexrazoxane and resveratrol, while no changes were observed in cells that received carvedilol. Although the 24 h pretreatment results revealed that all tested agents reduced ROS formation, no significant differences were observed between the cardioprotectants [45].

Therefore, the combination of antioxidant, anti-inflammatory, and vascular protective properties makes grape-derived polyphenols promising candidates for alleviating chemotherapy-associated cardiac damage.

### 4.3. The Pharmacokinetics and Metabolism of Grape Polyphenols

The pharmacokinetics of polyphenols such as resveratrol, monomeric phenols, and proanthocyanidins/oligomers vary and are determined by their chemical structure, degree of polymerization, and interaction with the gastrointestinal tract [115]. Studies of resveratrol in humans and its pharmacokinetic analysis consistently demonstrate substantial absorption after oral administration, but very low systemic bioavailability of the unchanged parent compound due to rapid presystemic metabolism, rapid formation of metabolites in the blood, and a short period of time during which the parent compound is eliminated from the body [116]. Gallic acid and isoflavones exhibit the highest oral bioavailability among dietary polyphenols, whereas catechins, flavanones, and quercetin glucosides are absorbed to a lesser extent and display distinct absorption and elimination kinetics [117]. However, it is important to distinguish between the absorption and systemic bioavailability of the unchanged parent compound. For instance, as described above for resveratrol, human pharmacokinetic studies indicate that resveratrol is substantially absorbed after oral administration, but the systemic bioavailability of the unchanged parent compound is very low because of rapid first-pass metabolism in the intestine and liver [118,119]. Therefore, low levels of unchanged resveratrol detected in blood circulation should not be interpreted as evidence of poor absorption.

When taken orally, some low molecular weight polyphenols can be absorbed in the small intestine, while most of the large condensed tannins and glycosides are not absorbed and pass into the large intestine [115]. In the large intestine, the gut microbiota plays a crucial role in determining the bioavailability of dietary polyphenols. There, they undergo extensive metabolism due to the action of microorganisms. This process generates metabolites such as phenylvalerolactones, phenylbutyric acid, etc., through enzymatic reactions, including deglycosylation, ring cleavage, dehydroxylation, and decarboxylation [120,121]. Many of these microbial metabolites are often more bioavailable in comparison with their parent compounds. They have higher absorption and may exert stronger biological activities [122]. For example, microbial metabolism of flavan-3-ols from red wine produces 3-hydroxyphenylpropionic acid, a metabolite that has been associated with cardioprotective effects [123].

Phenolic metabolites produced by gut bacteria are then either absorbed into the bloodstream or excreted in feces [121]. Following absorption, metabolites enter the liver through the portal vein, where they can undergo metabolic transformation, which generally comprises two phases. Phase I reactions are oxidation, reduction, and hydrolysis. These introduce or expose functional groups to increase the polarity of lipophilic molecules. However, most polyphenols typically undergo only limited phase I transformation and are predominantly subjected to phase II metabolism, including sulphation, methylation, glucuronidation, or a combination of some of the pathways [124]. Subsequently, new metabolites are distributed to organs or excreted in the urine [121]. Although phase II metabolism primarily occurs in the liver, conjugation reactions also take place in the small intestine [125].

Thus, the distinction between parent compounds and their metabolites is particularly important for the interpretation of experimental results. Future research should not focus exclusively on the biological activity of the initially administered polyphenols, but also on the transformations that occur following their ingestion and the therapeutic properties of the resulting metabolites. A more comprehensive approach would consider the efficacy of grape-derived polyphenols as a dynamic process, in which a spectrum of metabolites is formed and potentially contributes to the biological response.

The translational relevance of doses used in animal studies also requires consideration. Although in vivo studies provide evidence of the potential efficacy of different polyphenols, the doses administered experimentally may not be comparable with those achieved following oral administration in humans. Body surface area scaling (BSA) alone is not sufficient, as species differ substantially in intestinal absorption, first-pass metabolism, conjugation, tissue distribution, and elimination. For instance, Blanchard et al. highlighted the limitations of such an approach using resveratrol as an example. Based on previously reported pharmacokinetic data, they noted that 20 mg/kg in mice was believed to correspond to approximately 100 mg for a 60 kg human according to BSA conversion. However, as Blanchard et al. reported, a later clinical study using 100 mg resveratrol showed a substantially lower Cmax in humans (21.4 ng/mL) than that reported in mice (592 ng/mL), noting the limitations of relying on BSA-based dose conversion without considering pharmacokinetic differences between the two species [126]. Similar considerations apply to the concentrations used in in vitro studies. The 100 μM concentration of resveratrol used in some experiments is higher than the plasma concentrations of unchanged resveratrol typically reported following oral administration in humans [127]. Therefore, the biological effects observed at such concentrations should not be directly interpreted as evidence of clinically achievable exposure. However, the biological effects observed at such concentrations represent valuable evidence of the pharmacological potential of resveratrol and highlight the need to determine whether comparable effects can be achieved at clinically relevant concentrations and whether circulating or tissue metabolites contribute to these effects.

### 4.4. Safety and Potential Drug Interactions

Even though many studies report no adverse effects in healthy animals, these findings are not sufficient to establish the safety of grape polyphenol supplementation in patients with cancer. Oncology patients often have an increased risk of bleeding, hepatic or renal dysfunction, and may receive concomitant anticoagulant or antiplatelet therapy or undergo invasive procedures, all of which should be considered when evaluating the safety of polyphenol supplementation. Attention should be given to the reported ability of grape-derived polyphenols to inhibit platelet aggregation, as this effect may be beneficial for cardiovascular health [128]. Therefore, the potential clinical use of grape-derived polyphenols should consider each patient’s bleeding risk and concomitant medications.

Another important challenge is that the biological effects of grape-derived polyphenols may also strongly depend on the characteristics of the preparation. For instance, grape polyphenolic extracts can differ substantially in their polyphenol content because of factors including different plant parts, cultivars, extraction procedures, and manufacturing processes. Additionally, climate and seasonal weather strongly affect polyphenol content in grapes [129]. Therefore, there is an increased risk of batch-to-batch variability, which may further affect the efficacy of polyphenol supplementation. Studies using chemically characterized and standardized grape preparations are particularly important for their clinical translation.

Another important concern is the potential for pharmacokinetic interactions with concomitantly administered drugs. Polyphenols and their metabolites may affect enzymes responsible for drug metabolism and membrane transporters, including cytochrome P450 enzymes, P-glycoprotein, and organic anion-transporting polypeptides (OATPs) [127]. For instance, quercetin has been found to increase intracellular accumulation of doxorubicin and enhance its chemotherapeutic effect on breast cancer cells by downregulating efflux transporters, including P-glycoprotein, BCRP, and MRP1. Conversely, resveratrol may modulate CYP2D6-mediated metabolism of tamoxifen, resulting in decreased formation of endoxifen and thereby compromising therapeutic efficacy [130]. These findings highlight the need for caution when combining grape polyphenols with anticancer therapies. Further studies are required to obtain comprehensive data on interactions between chemotherapy drugs and grape-derived polyphenols.

Pharmacodynamic interactions between anticancer therapies and grape-derived polyphenols are another concern. Since oxidative mechanisms contribute to the cytotoxic effects of anticancer agents, such as doxorubicin, the antioxidant activity of grape-derived polyphenols might theoretically reduce the antitumor efficacy of administered drugs. However, the clinical relevance of such interactions remains insufficiently studied. This effect may depend on the specific polyphenol, its concentration, treatment schedule, and tumor characteristics. Research results often contradict each other. For instance, in a study by Henidi et al., quercetin demonstrated a contradictory dual effect. Quercetin protected against doxorubicin-induced vascular dysfunction and oxidative stress but simultaneously reduced its anticancer efficacy in a cell-line-dependent manner. The IC_50_ of doxorubicin in MCF-7 cells increased twofold in the presence of quercetin, with a combination index (CbnI) of 3.16, indicating antagonistic interaction. However, the combination of quercetin and doxorubicin did not induce any significant changes in the IC_50_ in MDA-MB-231 cells, with CbnI 2.04 (antagonistic). The opposite effect was found in T47D cells: coincubation with quercetin reduced the IC50 of doxorubicin from 0.7 ± 0.09 μM to 0.36 ± 0.05 μM, although the interaction was additive rather than synergistic (CbnI = 0.96) [131].

Unfortunately, information regarding pharmacokinetic or pharmacodynamic interactions between grape-derived polyphenols and trastuzumab is limited. Unlike anthracyclines, trastuzumab is a monoclonal antibody and is not metabolized by liver enzymes, making conventional CYP-mediated interactions unlikely. However, the potential influence of polyphenols on antibody disposition, immune-related mechanisms, and trastuzumab anticancer activity cannot be excluded.

## 5. Limitations and Prospects for the Use of Polyphenols

Polyphenols encounter challenges with bioavailability due to their susceptibility to external factors like heat, light, oxygen, and UV radiation, as well as internal factors such as stomach pH, solubility, diffusion, excretion, complex formation, hydrolysis, oxidation, and reduction. These factors result in low solubility, inadequate release, degradation, and reduced absorption. The use of polymeric materials for encapsulation addresses these issues by shielding polyphenols, enhancing their stability, and enabling controlled release, thus boosting bioavailability. Key findings indicate that encapsulating agents, including polymers, create a matrix that mitigates instability and facilitates improved dissolution, absorption, and pharmacological responses in areas such as pharmaceuticals and functional foods [132].

The absence of standardized doses and formulations of polyphenols represents a major challenge in research on their clinical effectiveness. A review of registered clinical trials by Marino et al. revealed considerable variability in dosages, forms (food products, extracts, isolated compounds), and the polyphenolic substances being studied, complicating the comparison of results across different studies and the identification of optimal usage regimens. Likewise, a review on the impact of polyphenols on bone health by Perrone et al. emphasizes that the lack of standardized formulations, dosages, and research protocols hinders the reliable evaluation of their therapeutic potential and weakens conclusions about their benefits. Overall, this inconsistency results in fragmented data that is difficult to synthesize for the development of clinical guidelines [133,134]. In future studies, it is important to focus on conducting thoroughly planned large-scale randomized clinical trials to verify the cardioprotective effects of natural dietary substances like polyphenols in patients undergoing anthracycline-based chemotherapy. Emphasis should be placed on enhancing bioavailability, establishing standardized dosages and formulations, and identifying optimal combinations and long-term safety profiles. This approach will facilitate the implementation of scientifically grounded strategies for utilizing nutraceuticals in cardiovascular medicine.

## 6. Conclusions

Chemotherapy-induced cardiotoxicity is a major challenge in the treatment of breast cancer, especially when anthracyclines and HER2-targeted therapies are applied. Even though these therapies have significantly improved survival rates, the adverse effects of chemotherapy agents pose a substantial risk of both acute and long-term cardiovascular complications. Cardiotoxicity is caused by several interconnected mechanisms, including oxidative stress, inflammation, and damage or death of cardiomyocytes.

The use of grape-derived polyphenols such as resveratrol, quercetin, catechins, and anthocyanins has been investigated as a potential strategy for attenuating adverse effects of chemotherapy-associated cardiotoxicity because of their diverse biological activities. Preclinical studies have demonstrated that polyphenols can reduce ROS, modulate signaling pathways, improve mitochondrial function, inhibit platelet aggregation, and exert other cardioprotective effects. However, these findings are derived mostly from experimental and preclinical models and should not be interpreted as confirmation of clinical benefit in patients. Therefore, grape-derived polyphenols represent promising candidates for further research as cardioprotective agents. However, it is important to note that this cardioprotective property has not been fully studied, posing a potential risk for translation into clinical practice. Several clinical studies have investigated grape-derived polyphenols in the context of breast cancer; however, most have focused on their effects on estrogen-related outcomes rather than on the prevention of chemotherapy-induced cardiotoxicity. To date, no completed clinical trials have specifically evaluated the cardioprotective efficacy of grape-derived polyphenols in patients with breast cancer receiving chemotherapy. However, a directly relevant clinical trial, NCT07419711, has been registered, but is currently ongoing, with no results yet available.

Clinical efficacy and safety remain unclear, and the available evidence does not support the routine recommendation of grape-derived polyphenol supplementation during anticancer therapy outside appropriately designed clinical studies. Therefore, there is an urgent need for well-designed randomized clinical trials to establish if grape-derived polyphenols can provide substantial cardioprotection without developing adverse effects.

Additionally, a crucial question that warrants further investigation is whether the administration of polyphenols can negatively impact the effectiveness of chemotherapy. Another limiting factor that needs to be addressed is their limited bioavailability. To overcome these barriers, recent advances have focused on innovative delivery systems that improve absorption and efficacy. Another significant difficulty is the lack of standardization, which prevents the translation of polyphenols into routine clinical practice. For instance, the variability in extraction methods, purification procedures, and quantitative determination of biologically active substances complicates the reproducibility and comparability of results. To address this issue, the widespread adoption of standardized protocols, such as those outlined in the WHO Good Agricultural and Collection Practices (GACP) for medicinal plants [135], is required. Furthermore, strict quality control and assurance comparable to pharmaceutical quality standards are crucial to ensuring product stability and patient safety. We emphasize these considerations as fundamental principles for the introduction of polyphenolic products into clinical practice.

## 7. Future Directions

Future research must be focused on addressing the main limiting factors, which are the lack of standardized dosages, the creation and implementation of administration protocols, and bioavailability. To address these limitations, novel delivery systems, such as phytosomes and plant-derived exosome-like nanoparticles, have been explored to enhance the bioavailability, stability, and targeted delivery of polyphenols. The latter are of particular interest since exosomes exhibit **a** wide range of therapeutic properties and naturally deliver different biologically active compounds from their parent plants [136]. Therefore, loading polyphenols into exosomes might not only help overcome the poor water solubility of polyphenols but also improve their therapeutic efficacy.

The effect of polyphenols on the efficacy of chemotherapy also requires careful evaluation to eliminate the risk of compromising its anticancer activity. In addition, long-term clinical studies are needed to better understand the therapeutic effects of polyphenol administration and to determine whether their use can reduce the incidence of late-onset cardiovascular complications that occur after chemotherapy is completed.

## Data Availability

No new data were created or analyzed in this study. Data sharing is not applicable to this article.

## Abbreviations

The following abbreviations are used in this manuscript:

AChE	Acetylcholinesterase
ADC	Antibody–Drug Conjugate
ADR	Adriamycin (Trade Name For Doxorubicin)
AH	Arterial Hypertension
AIC	Anthracycline-Induced Cardiotoxicity
ANP	Atrial Natriuretic Peptide
ATP	Adenosine Triphosphate
BSA	Body Surface Area
BNP	Brain Natriuretic Peptide
CAT	Catalase
CHF	Chronic Heart Failure
CI	Confidence Interval
CbnI	Combination Index
CK-MB	Creatine Kinase-MB
CNP	C-Type Natriuretic Peptide
COX	Cyclooxygenase
COX-1	Cyclooxygenase-1
CRP	C-Reactive Protein
Cu	Copper
CVD	Cardiovascular Disease
Cy	Cyanidin
DOCA	Deoxycorticosterone Acetate
Dp	Delphinidin
EF	Ejection Fraction
EMT	Epithelial–Mesenchymal Transition
ER	Estrogen Receptors
FS	Fractional Shortening
Fe	Iron
GA	Gallic Acid
GACP	Good Agricultural And Collection Practices
GPx	Glutathione Peroxidase
GR	Glutathione Reductase
GSH	Glutathione
GST	Glutathione S-Transferase
HDCT	High-Dose Chemotherapy
HDL	High-Density Lipoprotein
HER2	Human Epidermal Growth Factor Receptor 2
HF	Heart Failure
HIF-1α	Hypoxia-Inducible Factor 1 Alpha
HO-1	Heme Oxygenase-1
hs-CRP	High-Sensitivity C-Reactive Protein
hs-cTnI	High-Sensitivity Cardiac Troponin I
hs-cTnT	High-Sensitivity Cardiac Troponin T
i.g.	Intragastric
i.p.	Intraperitoneal
IHD	Ischemic Heart Disease
IL-1β	Interleukin-1 Beta
IL-6	Interleukin 6
I/R	Ischemia/Reperfusion Injury
LDL	Low-Density Lipoprotein
LDH	Lactate Dehydrogenase
LOX	Lipoxygenase
LV	Left Ventricle
LVEF	Left Ventricular Ejection Fraction
MD	Mean Difference
MDA	Malondialdehyde
MPO	Myeloperoxidase
Mv	Malvidin
NF-kB	Nuclear Factor Kappa B
NO	Nitric Oxide
NOS	Nitric Oxide Synthase
NT-proBNP	N-Terminal Pro-B-Type Natriuretic Peptide
NYHA	New York Heart Association
OATPs	Organic Anion-Transporting Polypeptides
POD	Peroxidase
Pg	Pelargonidin
Pn	Peonidin
PRs	Progesterone Receptors
Pt	Petunidin
ROS	Reactive Oxygen Species
SIRT1	Sirtuin 1
SOD	Superoxide Dismutase
TAC	Total Antioxidant Capacity
TNBC	Triple-Negative Breast Cancer
TnI	Troponin I
Top2β	Topoisomerase Iiβ
TOS	Total Oxidant Status
VEGF	Vascular Endothelial Growth Factor
WHO	World Health Organization
Zn	Zinc
